# Validation of total skin electron irradiation (TSEI) technique dosimetry data by Monte Carlo simulation

**DOI:** 10.1120/jacmp.v17i4.6230

**Published:** 2016-07-08

**Authors:** Alexander Nevelsky, Egor Borzov, Shahar Daniel, Raquel Bar‐Deroma

**Affiliations:** ^1^ Oncology, Rambam Health Care Campus Haifa Israel

**Keywords:** total skin electron irradiation, Monte Carlo simulation, dosimetry

## Abstract

Total skin electron irradiation (TSEI) is a complex technique which requires many nonstandard measurements and dosimetric procedures. The purpose of this work was to validate measured dosimetry data by Monte Carlo (MC) simulations using EGSnrc‐based codes (BEAMnrc and DOSXYZnrc). Our MC simulations consisted of two major steps. In the first step, the incident electron beam parameters (energy spectrum, FWHM, mean angular spread) were adjusted to match the measured data (PDD and profile) at SSD=100 cm for an open field. In the second step, these parameters were used to calculate dose distributions at the treatment distance of 400 cm. MC simulations of dose distributions from single and dual fields at the treatment distance were performed in a water phantom. Dose distribution from the full treatment with six dual fields was simulated in a CT‐based anthropomorphic phantom. MC calculations were compared to the available set of measurements used in clinical practice. For one direct field, MC calculated PDDs agreed within 3%/1 mm with the measurements, and lateral profiles agreed within 3% with the measured data. For the OF, the measured and calculated results were within 2% agreement. The optimal angle of 17° was confirmed for the dual field setup. Dose distribution from the full treatment with six dual fields was simulated in a CT‐based anthropomorphic phantom. The MC‐calculated multiplication factor (B12‐factor), which relates the skin dose for the whole treatment to the dose from one calibration field, for setups with and without degrader was 2.9 and 2.8, respectively. The measured B12‐factor was 2.8 for both setups. The difference between calculated and measured values was within 3.5%. It was found that a degrader provides more homogeneous dose distribution. The measured X‐ray contamination for the full treatment was 0.4%; this is compared to the 0.5% X‐ray contamination obtained with the MC calculation. Feasibility of MC simulation in an anthropomorphic phantom for a full TSEI treatment was proved and is reported for the first time in the literature. The results of our MC calculations were found to be in general agreement with the measurements, providing a promising tool for further studies of dose distribution calculations in TSEI.

PACS number(s): 87.10. Rt, 87.55.K, 87.55.ne

## I. INTRODUCTION

Total skin electron irradiation (TSEI) is one of the most effective treatments of malignant skin diseases, such as mycosis fungoides (MF) and cutaneous lymphomas^1^, ^2^, ^3^, ^4^ The goal is to treat the entire body surface to a limited depth with a relatively uniform dose (e.g., ±10%).^(5)^ In order to deliver the treatment dose at a shallow depth, low‐energy electrons are considered for TSEI, which is commonly performed with a 6 MeV beam. Over the years, various techniques have been developed for TSEI; their descriptions can be found in the AAPM Report 23, “Total Skin Electron Therapy: Technique and Dosimetry”.^(6)^ According to the recommendations of this report, a uniform dose distribution in an area of 200 cm in height and 80 cm in width should be available. In addition, bremsstrahlung X‐ray contamination should be low and the dose rate at the patient should be high (at least 0.25 Gy/min, practically over 1 Gy/min).

To achieve these recommendations, we use the Stanford technique developed by Karzmark and colleagues.^(7)^ This technique has been considered as a safe and effective treatment, and has been selected by a majority of institutions in the last 20 years as the preferred modality.^(2)^ The Stanford technique is characterized by the use of: 1) an extended SSD (more than 3 m); 2) dual large electron fields with central axis angled by approximately $\pm 20^{\circ }$ from the horizontal; and 3) six patient positions relative to each of the two large angled TSEI beams (anterior, posterior, and four lateral obliques).

TSEI is a complex technique which requires a large number of nonstandard measurements and dosimetric procedures. To validate the measured dosimetric data, Monte Carlo (MC) simulations can be used. It is well recognized that MC dose calculations are the most accurate way of computing relative dose distributions in radiotherapy. However, the application of MC for TSEI beams has been limited, primarily because it involves time‐consuming, large‐scale electron transport simulations.

Pavón et al.^(8)^ applied the MC technique to evaluate dosimetric properties of one large TSEI beam by full‐modeling their linear accelerator (linac) using the EGS4 code. Ye et al,^(9)^ employed the MC technique to design the optimal scattering foil and to estimate patient doses in both stationary and rotational setups for TSEI. In their work, the EGS4 code was used to build a full model of their linac and to calculate dose distributions in a cylindrical phantom under TSEI treatment conditions. Faj et al.^(10)^ employed a simplified multiple‐source MC model for electron beams and tested it by comparing calculations and experimental results in a cylindrical phantom for the full TSEI treatment using the Stanford technique (six dual fields).

The purpose of this work is to validate the measured dosimetry data by MC simulations. MC simulations of dose distributions from single and dual fields at the treatment distance were performed in a water phantom. Dose distribution from the full treatment with six dual fields was simulated in a CT‐based anthropomorphic phantom. MC calculations were compared to the available set of measurements used in clinical practice.

## II. MATERIALS AND METHODS

### A. TSEI technique

The 6 MeV beam from the Elekta Precise linac (Elekta AB, Stockholm, Sweden) operated in high‐dose‐rate electron (HDRE) mode (dose rate about 30 Gy/min at the isocenter) is used for TSEI treatments, based on the Stanford technique, in our department. The HDRE insert is electronically interlocked to open the field size to its maximum dimensions of $40\times 40\;{\text{cm}}^{2}$ at 100 cm SSD. The patient stands at 400 cm SSD. A 0.5 cm PMMA plate (a degrader) may be placed at 30 cm in front of the patient. This plate provides energy degradation of the electron beam and is used in situations where the disease is superficial. The degrader also provides additional large‐angle scattering and dose homogeneity.

## B. Measurements

Measurements for beam characterization were performed at 100 cm SSD in the HDRE mode. The depth dose along the central axis and lateral profiles were measured in a water phantom (MP3, PTW, Freiburg, Germany) using the EFD electron diode (Scanditronix, Uppsala, Sweden). Output in terms of Gy per MU was measured in the same water phantom using a plane‐parallel Roos PTW ion chamber (PTW, Freiburg, Germany).

At the treatment distance, the measurements were performed in a RMI‐457 Solid Water phantom (Gammex RMI, Middleton, WI) using the Roos chamber. The PDD curve was obtained by adding plates of various thicknesses while maintaining the SSD at 400 cm. The lateral profile was measured at 1 mm depth by shifting the Solid Water phantom housing horizontally.

The absolute dose per 100 MU for one straight field (gantry angle at 90°) was measured at the depth of maximal dose (1.2 cm without degrader and 0.7 cm with degrader) at SSD 400 cm. These conditions are further referenced as the calibration conditions. The output factor (OF) was calculated as the ratio of dose at SSD=400 cm to dose at SSD=100 cm, both for the measurements and for the MC simulation. As large portions of ion chamber cable are irradiated during TSEI measurements, the cable effect for the Roos chamber was checked and was found negligible (less than 0.3%).

The optimal angle defined by the MC calculations was verified by measurements of the vertical profile from a single field and from a dual field using thermoluminescent dosimeter (TLD) chips (Harshaw TLD‐100). The TLDs were read out using a Harshaw 3500 TLD reader (Thermo Fisher Scientific Inc., Waltham, MA).

A 2D transversal dose distribution of the whole treatment (six dual fields) was measured in the anthropomorphic RANDO phantom (The Phantom Laboratory, Salem, NY) using film dosimetry. The phantom with Gafchromic film EBT2 (ISP, Wayne, NJ) placed inside was irradiated under clinical TSEI conditions. An Epson 1000XL flatbed scanner (Epson America, Inc., Long Beach, CA) and ImageJ software (NIH, Bethesda, MD) were used to digitize and analyze the films.

The multiplication factor (B12‐factor), which relates the skin dose for the whole treatment to the dose from one calibration field, was calculated from measurements with TLD detector chips placed on the surface of the anthropomorphic phantom. A phantom setup was made identical to the treatment setup by changing the positions every 60° about the phantom's longitudinal axis. TLD chips were attached to the phantom surface in the abdominal section. The measured B12‐factor was defined as the ratio of the mean TLD reading from the whole treatment to the TLD reading in the calibration conditions from one field.

Finally, X‐ray contamination was defined as the dose in the phantom at the depth behind the practical range divided by the treatment skin dose. X‐ray contamination from one field was measured with the Roos ion chamber in the Solid Water phantom at the depth of 5 cm. The point of measurements was at the horizontal central axis while the gantry was rotated by 17°. The measured full treatment X‐ray contamination was estimated as the measured X‐ray contamination from one rotated field multiplied by 12 (total number of fields).

### C. MC simulations

All MC simulations in this study were performed with the EGSnrc code^(11)^ for coupled electron and photon transport. EGSnrc is an improved version of the EGS4 code.^(12)^ This code has been extensively validated in the past and seemed best suited for our study.

BEAMnrc^(13)^ is an EGSnrc‐based package that allows for the simulation of radiotherapy treatment units using predefined component modules (CM). BEAMnrc code produces phase‐space files containing all the necessary information characterizing every particle at a specified scoring plane. The phase‐space data can then be used as an input to calculate dose distributions in water phantoms or CT‐based phantoms.

The DOSXYZnrc^(14)^ code can be used to calculate the three‐dimensional (3D) absorbed radiation dose distributions in either a rectangular Cartesian phantom with any arbitrary voxel distribution with dimensions and material composition defined by user for each voxel, or in a 3D virtual phantom based on a CT scan. In both cases, the geometry and material composition have to be predefined before the calculations. DOSXYZnrc simulates particle transport in a 3D volume and scores the deposited energy in all designated voxels. The output of a DOSXYZnrc calculation is a file that stores the calculated 3D dose distribution data and the corresponding dose uncertainties.

**Figure 1:**
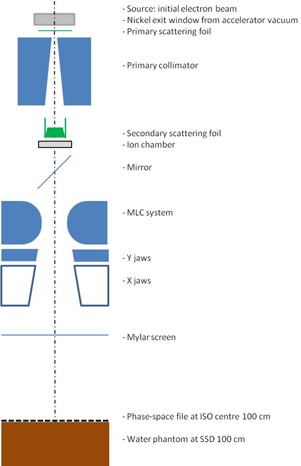
Modeling geometry at SSD=100 cm including Elekta Precise components, water phantom, and the phase‐space location.

The modeled geometry for the Elekta Precise linac was entered into the BEAMnrc code using full details of the linac head provided by the manufacturer. Only parts exposed to the radiation beam were modeled. The schematic diagram of the MC model based on an Elekta Precise is shown in Fig. 1. In our work, the linac geometry for the 6 MeV beam consists of the exit window, the primary scattering foils, the primary collimator, the secondary scattering foils, the ion chamber, the mirror, the MLC, the upper jaws (called the back‐up jaws in Elekta terminology) and the lower jaws (called the X jaws in Elekta terminology), the Mylar, and the air slab (needed to specify the position of the space‐phase file).

Following several published studies,^8^, ^15^, ^16^ the cutoff energies $P_{\text{cut}}$ and $E_{\text{cut}}$ were set to 0.01 MeV and 0.7 MeV, respectively. For all simulations, the boundary crossing algorithm was EXACT and the electron step algorithm was PRESTA‐II. The user‐adjustable values for other parameters were set at their default values. In order to guarantee good statistical accuracy of dose simulations, up to 10^9^ primary electrons were transported from the exit window, depending on the simulation geometry.

Our MC simulations consisted of two major steps. First, the incident electron beam parameters (energy spectrum, FWHM, mean angular spread) were adjusted to match the measured data (PDD and profile) at SSD=100 cm for an open field. These parameters were then used to calculate dose distributions at the treatment distance of 400 cm. BEAMnrc code was used to generate the phase‐space file in a plane at SSD=100 cm. This file was used in the DOSXYZnrc code to calculate PDDs, profiles, and output from a single beam in a water phantom at SSD=100 cm with a voxel size of $0.5\times 0.5\times 0.2\;{\text{cm}}^{3}$ (Fig. 1). Statistical uncertainties of the calculated doses in the region of interest were less than 1%.

**Figure 2:**
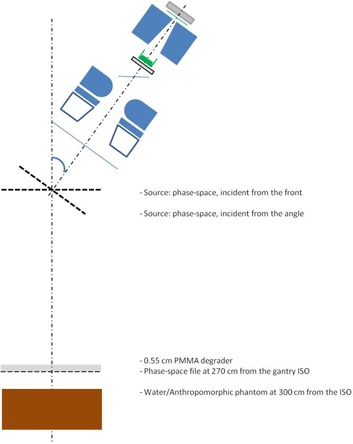
Modelling geometry at SSD=400 cm. Linac components and location of the degrader, solid water phantom, and the phase‐space file are shown.

For calculations at the treatment distance, the phase‐space file at SSD=100 cm was used as a source within the BEAMnrc code, and a second phase‐space file was created at SSD=370 cm (immediately after the PMMA degrader). For the dual field, the phase‐space file at SSD=100 cm was first rotated by the dual‐field angle at the isocenter plane and used as a source for the creation of the second phase‐space file at SSD=370 cm (Fig. 2).

The second phase‐space file was then used in the DOSXYZnrc code for dose calculations at SSD=400 cm in a water phantom with a voxel size of $3\times 3\times 0.2\;{\text{cm}}^{3}$ and in a CT‐based phantom with voxel size of $0.3\times 3\times {\text{cm}}^{3}$. Dose distributions were calculated with statistical uncertainties of less than 1% in the region of interest.

For the MC‐calculated and the measured data comparison, the following methods were used: PDDs and profiles agreement at SSD=100 cm was evaluated using a gamma method with 2%/1 mm criteria, PDDs agreement at SSD=400 cm was evaluated using a gamma method with 3%/1 mm criteria, and profiles agreement at SSD=400 cm was evaluated using dose difference test with 3% criteria.

The degrader was modeled within BEAMnrc as a layer of PMMA voxels with corresponding density. This is due to the fact that the DOSXYZnrc does not allow definition of additional voxels apart from those defined by the CT; thus, one cannot model a degrader plate together with a CT‐based phantom.

To define the optimal gantry angle for the dual field, vertical profiles at SSD=400 cm and 0.25 cm depth were calculated for gantry angles tilted by 15°, 16°, 17°, 18°, and 19° from the horizontal central axis. Then the profile was mirrored relative to the central axis and a total profile (the sum of two profiles) was created. An optimal angle would give the most uniform total profile.

In order to calculate dose distribution from the whole treatment in the RANDO phantom, one has to generate a DOSXYZ phantom from the phantom CT data first. This is done using the CTCREATE user code, with the relationship between Hounsfield units (HU) and density determined via linear interpolation between a series of specified points on the “CT‐density ramp”. Tissue types are assigned according to HU ranges. Each voxel in the DOSXYZnrc phantom, therefore, has an electron density (electrons/cm^3^) defined by the product of the mass density (from the HU conversion) and the intrinsic electron density (electrons/gram) from the material assignment in that voxel. In our study, we used the default CT‐density ramp and four types of materials with the corresponding electron densities — air, soft tissue, bone, and lung — for the material assignment. To overlay the isodose contours on the CT slices, we used the DOSXYZ_SHOW code.

The treatment skin dose has been defined as the mean dose at or near the surface of the anthropomorphic phantom which has been irradiated with all six dual fields. The MC calculated B12‐factor was defined as the ratio of the mean surface dose in the abdominal section received when all 12 fields are delivered to the dose received from one calibration field, all fields receiving the same number of MUs.

MC‐calculated X‐ray contamination was defined as the mean dose in the inner region of the anthropomorphic phantom (behind the practical range) divided by the treatment skin dose.

## III. RESULTS AND DISCUSSION

**Figure 3:**
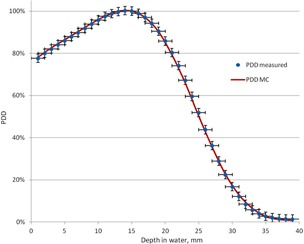
The measured and calculated PDD curves for a $40\times 40\;{\text{cm}}^{2}$ field at SSD=100 cm.

**Figure 4:**
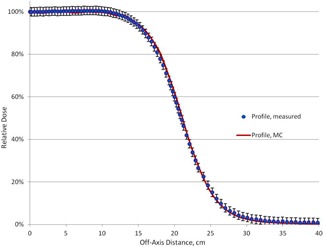
The measured and calculated lateral profiles at depth = 1.2 cm for a $40\times 40\;{\text{cm}}^{2}$ field at SSD=100 cm.

The measured and calculated PDD curves for a $40\times 40\;{\text{cm}}^{2}$ field at SSD=100 cm are shown in Fig. 3. Lateral profiles measured and calculated in these conditions at 1.2 cm depth are shown in Fig. 4. It can be seen that all calculated and measured results agree within 2%/1 mm. The primary electron beam incident on the foil was assumed to have a mean angular spread of 1.5° and to be distributed within a circular area having Gaussian distribution of 0.11 cm FWHM. The energy distribution of the primary electron beam was assumed to be Gaussian with a mean energy of 7.2 MeV and FWHM of 2.8 MeV. This distribution was truncated at the mean energy ±1.0 MeV.

**Figure 5:**
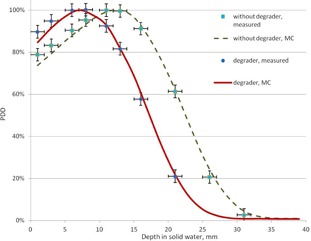
The measured and calculated PDD curves (with and without degrader) at SSD=400 cm.

**Figure 6:**
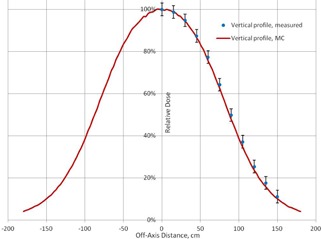
The measured and calculated profiles at depth 0.25 cm at SSD=400 cm.

At the treatment distance, the PDD curves and lateral profiles obtained with the MC calculations are compared with the measured data in Figs. 5 and 6. It can be seen that the MC‐calculated PDD curves agree to within 3%/1 mm with the measured data for all points; for the lateral profiles, all points agree within 3%.

**Figure 7:**
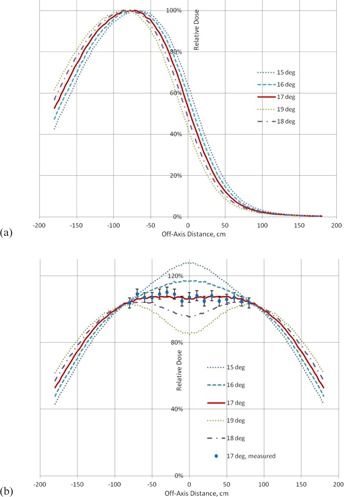
The calculated profiles at depth 0.25 cm at SSD=400 cm for: (a) one field for different tilt angles, and (b) different dual‐field angles. For the 17° angle, measurements at depth 0.25 cm are also shown.

Lateral profiles calculated for different tilt angles and corresponding total dual‐field vertical profiles are presented in Fig. 7. As the most uniform profile is obtained with a 17° tilt, it is selected to be the optimal dual‐field angle. For this angle, profile uniformity is within ±3% over 160 cm height, compared with the vertical uniformity requirement of ±8% defined by the AAPM Report 23.^(6)^ The measured data for a dual field with 17° angle have been added in Fig. 7.

**Figure 8:**
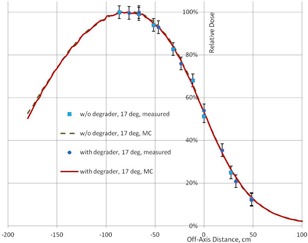
The calculated and measured profiles at depth 0.25 cm for one field with 17° tilt, with and without degrader, at SSD=400 cm.

The use of a degrader does not affect the shape of lateral profile, and the optimal angle remains the same; this is demonstrated in Fig. 8.

**Figure 9:**
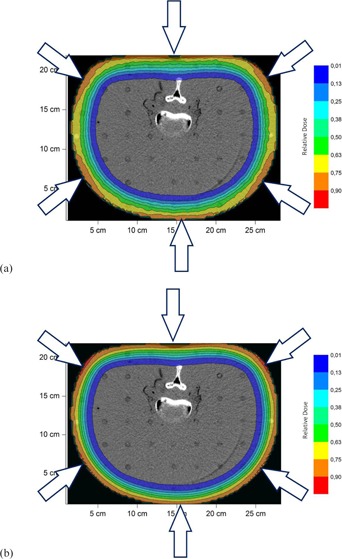
The MC‐calculated transversal dose distribution from the whole treatment in the anthropomorphic phantom: (a) no degrader is used; (b) the degrader is used. Treatment fields are shown with the arrows.

The MC‐calculated transversal dose distribution from the whole treatment in the anthropomorphic phantom is shown in Fig. 9. Looking at this figure it can be concluded that a degrader provides more homogeneous dose distribution.

**Figure 10:**
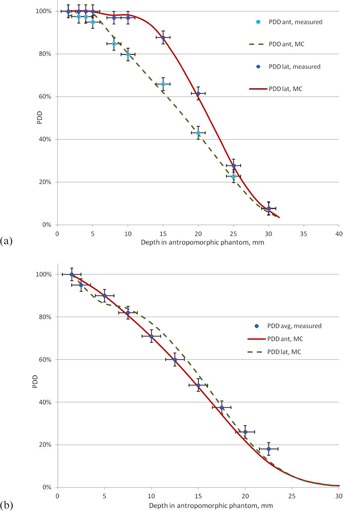
MC‐calculated and measured PDDs along anterior and lateral directions in the anthropomorphic phantom: (a) no degrader; (b) with degrader.

Figure 10 presents a comparison of MC‐calculated and measured (with film dosimetry) PDD curves taken along anterior and lateral directions. This comparison confirms that, when the degrader is used, the difference between anterior and lateral PDDs becomes small, which means a more homogeneous dose distribution.

Output factors' values obtained by the measurements with and without the degrader were $5.2\times 10^{-2}$ and $5.3\times 10^{-2}$, respectively. Corresponding output factors obtained from the MC calculation were $5.3\times 10^{-2}$ and $5.4\times 10^{-2}$. Comparing these results, one can observe a < 2% deviation between the values of output factors obtained by measurements and MC calculation. This difference includes both the experimental and calculation uncertainties, which are within an order of magnitude of the deviation between both results. If we calculate the OF based on the inverse square law only, the result would be ${\left(1/4\right)}^{2}=6.25\times 10^{-2}$. It can be seen that the measured and calculated results for OF are slightly lower compared to the inverse square prediction; this is due to additional scatter of electrons in air. The same effect was observed previously.^(8)^

The MC‐calculated B12‐factor for setups with and without degrader was 2.9 and 2.8, respectively. The measured B12‐factor was 2.8 for both setups. The difference between calculated and measured values was within 3.5%.

The measured X‐ray contamination for the full treatment was 0.4%; this is compared to the 0.5% X‐ray contamination obtained with the MC calculation. The measured and MC‐calculated results for X‐ray contamination were in good agreement; as recommended, the X‐ray contamination was less than 1%.^(5)^

## V. CONCLUSIONS

The results of our Monte Carlo calculations were found to be in general agreement with the measurements, providing a promising tool for further studies of dose distribution calculations in TSEI. The measured dosimetry data used for TSEI calculations were validated by MC simulations. To the best of our knowledge, this is the first time that a full TSEI treatment simulation in an anthropomorphic phantom is reported. Feasibility of MC simulation in an anthropomorphic phantom for a full TSEI treatment was proved. This work also indicates that simulations can complement and/or replace extensive experimental measurements needed for the commissioning of TSEI techniques.

## COPYRIGHT

This work is licensed under a Creative Commons Attribution 3.0 Unported License.
